# Retinal Structural and Microvascular Findings on OCT and OCTA in Hemodialysis and Kidney Transplant Recipients and Their Association with Mineral Metabolism Parameters

**DOI:** 10.3390/medsci14030381

**Published:** 2026-07-09

**Authors:** Ioana-Mădălina Bîlha, Ștefana Cătălina Bîlha, Nada Akad, Adrian Covic, Daniel-Constantin Brănișteanu, Simona Hogaș, Mihai Marian Hogaș, Anca Matei, Maria-Christina Ungureanu, Calina Anda Sandu-Boz, Camelia Margareta Bogdănici, Irina Draga Căruntu

**Affiliations:** 1 Department of Morpho-Functional Sciences I-Histology, “Grigore T. Popa” University of Medicine and Pharmacy, 700115 Iasi, Romania; ioana-madalina.bilha@email.umfiasi.com (I.-M.B.); irina.caruntu@umfiasi.ro (I.D.C.); 2 Department of Internal Medicine II-Endocrinology, “Grigore T. Popa” University of Medicine and Pharmacy, 700115 Iasi, Romania; anca.matei@umfiasi.ro (A.M.); maria.ungureanu@umfiasi.ro (M.-C.U.); 3 Department of Internal Medicine II-Nephrology, “Grigore T. Popa” University of Medicine and Pharmacy, 700115 Iasi, Romania; akad_nada@d.umfiasi.ro (N.A.); adrian.covic@umfiasi.ro (A.C.); simona.hogas@umfiasi.ro (S.H.); 4 Department of Surgery II-Ophthalmology, “Grigore T. Popa” University of Medicine and Pharmacy, 700115 Iasi, Romania; daniel.branisteanu@umfiasi.ro (D.-C.B.); calina-anda.sandu@umfiasi.ro (C.A.S.-B.); camelia.bogdanici@umfiasi.ro (C.M.B.); 5 Department of Morpho-Functional Sciences II, Discipline of Physiology, “Grigore T. Popa” University of Medicine and Pharmacy, 700115 Iasi, Romania; mihai.hogas@umfiasi.ro; 6 Romanian Medical Science Academy, 030171 Bucharest, Romania

**Keywords:** chronic kidney disease, hemodialysis, renal transplantation, optical coherence tomography angiography, foveal avascular zone, retinal microvasculature, mineral metabolism

## Abstract

Background: Chronic kidney disease (CKD) is associated with systemic microvascular dysfunction, yet the relationship between retinal changes and mineral metabolism disturbances across CKD treatment modalities remains incompletely characterized. Methods: We performed a baseline cross-sectional analysis of 77 participants (29 hemodialysis [HD] patients, and 48 kidney transplant recipients [KTR]) who underwent 7 × 7 mm structural optical coherence tomography (OCT) and 6 × 6 mm OCT angiography (OCTA) imaging. Structural parameters included central macular thickness (CMT), ganglion cell layer plus inner plexiform layer (GCL+), ganglion cell complex (GCL++), and subfoveal choroidal thickness (SCT). Microvascular parameters included superficial and deep capillary plexus vessel density (SVPD, DVPD), choriocapillaris density (CCD), and foveal avascular zone (FAZ) area. Serum mineral metabolism markers were correlated with retinal findings. Eyes were analyzed separately as the primary approach, while generalized estimating equation (GEE) models adjusted for age, sex, body mass index, diabetes, and hypertension (HTN) were used for confirmation. Results: FAZ area, SVPD, DVPD, CCD, GCL+, GCL++, and CMT did not differ significantly between cohorts in either per-eye analyses or adjusted GEE models. SCT was lower in HD patients than in KTR in the right eye (*p* = 0.019) and remained significantly higher in KTR in the adjusted GEE models (*p* = 0.016), a difference that persisted after excluding participants with diabetes and after adjustment for HTN grade. HTN grade was associated with FAZ area in HD and SCT in KTR. Serum magnesium was inversely associated with FAZ area in HD (B = −95.27, *p* = 0.034) and positively associated with GCL+ in KTR (B = 10.55, *p* = 0.048). Conclusions: Retinal microvascular and inner-retinal structural parameters were largely comparable between HD patients and KTR, with SCT the only parameter showing a consistent between-cohort difference (higher in KTR). Serum magnesium and HTN grade were the metabolic and clinical variables most consistently associated with retinal measures.

## 1. Introduction

Chronic kidney disease (CKD) is widely regarded as a systemic microvascular condition, characterized by endothelial dysfunction, oxidative stress, persistent inflammation, and dysregulation of mineral metabolism. Progressive peritubular capillary rarefaction, endothelial glycocalyx degradation, and vascular calcification collectively amplify systemic vascular injury in advanced CKD [1,2,3,4], and these alterations frequently extend to distant vascular beds, including the retinal circulation [5].

The retinal circulation shares important anatomical and physiological features with the renal and cerebral microvasculature, potentially offering a non-invasive window into systemic vascular integrity. Advances in optical coherence tomography angiography (OCTA) allow high-resolution, layer-specific visualization of the superficial and deep retinal capillary plexuses, quantitative assessment of the foveal avascular zone (FAZ), and evaluation of choroidal perfusion metrics [5,6,7,8,9]. Our group recently published a systematic review summarizing the current evidence on OCTA changes in CKD [9]. Previous studies have reported alterations in macular and optic disk perfusion in hemodialysis (HD) patients, as well as changes in subfoveal choroidal thickness (SCT) and retinal microcirculation across CKD stages [10,11]. However, most available studies compare CKD patients with healthy controls, and few have examined retinal changes across different renal replacement modalities or whether these alterations are modified following renal transplantation [12,13].

Beyond their skeletal consequences, CKD-related mineral and bone disorders (CKD-MBD) have notable vascular effects: elevated parathyroid hormone (PTH) levels have been associated with endothelial dysfunction and vascular stiffness [14], hyperphosphatemia has been linked to vascular calcification [4], and hypomagnesemia has been associated with impaired endothelial function and oxidative stress [15,16]. Given that the retinal microvasculature shares structural and functional properties with other end-organ microvascular beds, mineral metabolism abnormalities may plausibly contribute to retinal microvascular and neuronal alterations [17]. However, this relationship has not been systematically explored across renal replacement modalities.

The aim of the present study was to evaluate retinal microvascular and structural parameters using OCT and OCTA in chronic HD patients and kidney transplant recipients (KTR) and to explore potential associations between mineral metabolism parameters and retinal findings across these groups.

## 2. Materials and Methods

### 2.1. Study Design

We performed a cross-sectional, observational study using interdisciplinary baseline data to evaluate OCT and OCTA retinal changes and their relationship with mineral metabolism in patients with CKD. A one-year prospective follow-up of this cohort is ongoing and will be reported separately.

The primary objective was to compare retinal structural (OCT) and microvascular (OCTA) parameters between chronic HD patients and KTR. The secondary objective was to explore associations between these retinal parameters and markers of mineral metabolism and renal function within each group.

HD patients aged 20–70 years who had been on maintenance HD for at least one year were included, irrespective of vascular access type or dialysis modality. To minimize acute hemodynamic fluctuations, all HD patients were examined on a non-dialysis day. KTR aged 20–70 years with a functioning graft at least one year after transplantation were included, irrespective of donor type, pre-transplant dialysis history, or immunosuppressive regimen. Dialysis vintage was documented in the HD group. In KTR, time since transplantation, duration of prior HD, type of immunosuppressive therapy and cumulative glucocorticoid (GC) dose were recorded. The requirement of at least one year of maintenance HD or graft function was chosen to capture a stable treatment state and to avoid the confounding effects of the early peri-dialysis or peri-transplant period (acute fluid shifts, unstable graft function, and high-dose induction immunosuppression). Finally, CKD etiology and antihypertensive medication and use of statins were recorded in both groups.

Common exclusion criteria for both groups were pregnancy or lactation; active pharmacological treatment for osteoporosis other than calcium and vitamin D supplementation; current or previous malignancy; liver cirrhosis; recent major fractures; prior non-renal organ transplantation; and secondary causes of osteoporosis unrelated to CKD or transplantation. HD patients were additionally excluded if they had previously received a kidney graft, and KTR were excluded in cases of return to dialysis or acute graft rejection.

HD patients and KTR were consecutively recruited from CKD patients followed in the Nephrology Department over a 12-month period. Five hundred and fifty KTR and 290 HD patients were invited to participate. After exclusion of those who did not fulfill the study criteria or declined to take part, 48 KTR and 29 HD patients were enrolled. Written informed consent was obtained from all participants prior to inclusion.

All participants underwent a comprehensive evaluation including a detailed history cross-checked with medical records, physical examination, hypertension (HTN) status, fasting morning blood sampling for serum determinations, and bilateral OCT and OCTA imaging during a single study visit. A representative OCTA acquisition, including segmentation boundaries, is provided in Supplementary Figure S1.

### 2.2. Biological Profile

Laboratory parameters included serum creatinine, estimated glomerular filtration rate (eGFR), albumin-corrected serum calcium, phosphate, magnesium, PTH, and 25-hydroxyvitamin D. Serum intact PTH was measured by electrochemiluminescence immunoassay (ECLIA) and 25-hydroxyvitamin D by electrochemiluminescence binding assay, using a Roche cobas^®^ E601 immunoassay analyzer (Roche, Branchburg, NJ, USA). Serum calcium, phosphate, magnesium, and creatinine concentrations were determined by photometric/colorimetric methods. Albumin-corrected serum calcium was calculated as: corrected calcium (mg/dL) = total calcium (mg/dL) + 0.8 × [4.0 − serum albumin (g/dL)] [18]. The validated CKD-EPI equation was used to estimate eGFR [19].

### 2.3. Ophthalmologic Examination/OCT and OCTA Imaging Protocol

All participants underwent a comprehensive ophthalmologic examination in the Ophthalmology Department, including best-corrected visual acuity (BCVA) measurement, intraocular pressure (IOP) assessment, slit-lamp biomicroscopy, and dilated fundus examination. Both eyes were included in the analysis.

All OCT and OCTA examinations were performed by a single experienced operator using a DRI OCT Triton swept-source device (Topcon, Tokyo, Japan) with IMAGEnet 6 software (version 2.53), incorporating the proprietary OCT Angiography Ratio Analysis (OCTARA) algorithm. The imaging protocol included a 7 × 7 mm macular cube scan for structural evaluation and a 6 × 6 mm OCTA scan centered on the fovea for vascular assessment.

Structural parameters included CMT, SCT, macular retinal nerve fiber layer (mRNFL) thickness, GCL+ (ganglion cell layer plus inner plexiform layer), and GCL++ (ganglion cell complex).

OCTA acquisitions enabled quantification of perfusion density within the superficial vascular plexus (SVP), deep vascular plexus (DVP), and choriocapillaris (CC). According to the device segmentation algorithm, SVP density (SVPD) was calculated from 2.6 µm below the internal limiting membrane (ILM) to 15.6 µm below the IPL/INL junction, while DVP density (DVPD) was measured from 15.6 µm to 70.2 µm below the IPL/INL junction. The choriocapillaris slab was automatically segmented at the level of the outer retina/choriocapillaris interface, in accordance with the device default settings.

Because the device does not provide automated FAZ quantification, the FAZ area was manually delineated on en face OCTA images at the level of the SVP using the built-in caliper tool. All measurements were performed by a single examiner masked to group allocation. Intra-observer reproducibility was assessed in 20 randomly selected eyes using repeated measurements and demonstrated excellent agreement; the intraclass correlation coefficient for FAZ area was 1.000 (95% CI, 1.000–1.000) in both eyes.

### 2.4. Study Endpoints

The primary outcomes were between-group differences in OCTA microvascular parameters (SVPD, DVPD, CC density, and FAZ area) and structural OCT parameters (CMT, SCT, mRNFL thickness, GCL+, and GCL++). Secondary outcomes were the associations between these retinal parameters and markers of mineral metabolism (PTH, corrected serum calcium, phosphate, magnesium, and 25-hydroxyvitamin D) and renal function (eGFR).

### 2.5. Statistical Analysis

Statistical analyses were performed using IBM SPSS Statistics for Macintosh, version 29.0 (IBM Corp., Armonk, NY, USA). Because this was an exploratory, hypothesis-generating study based on consecutive enrolment within a fixed 12-month window, no a priori sample-size calculation was performed. To contextualize the achieved sample for the primary two-cohort comparison, we performed a post hoc sensitivity analysis. With α = 0.05 (two-sided) and 80% power, the available renal cohort sample (HD = 29, KTR = 48) was adequate to detect moderate-to-large between-group differences, corresponding to Cohen’s d ≈ 0.67 for the HD-versus-KTR comparison. For within-group correlation analyses, the detectable effect size was approximately |r| ≈ 0.49–0.50 in the HD group and |r| ≈ 0.39–0.40 in the KTR group. The study was therefore underpowered to detect small between-group differences or weak within-group correlations, and borderline or unilateral findings should be interpreted in this context.

The distribution of continuous variables was assessed using the Shapiro–Wilk test, and homogeneity of variances was evaluated using Levene’s test where appropriate. Continuous data are presented as mean ± standard deviation (SD). For comparisons between two groups, the independent-samples Student’s *t*-test was used for normally distributed variables and the Mann–Whitney U test for non-normally distributed variables.

Associations between continuous variables were evaluated using the Pearson correlation coefficient for normally distributed variables and the Spearman rank correlation coefficient for non-normally distributed variables. Significant correlations were further entered into bivariate regression analysis to quantify the association between pairs of variables. For ocular parameters that showed significant correlations with more than one systemic or clinical variable within the same study group, multiple linear regression models were fitted to assess independent associations. Adjusted models were then fitted to account for potential confounding.

Generalized estimating equation (GEE) models were used as the main adjusted analysis to account for within-subject inter-eye correlation. Each retinal outcome was modeled as the dependent variable, with study group as the main independent variable and age, sex, BMI, diabetes mellitus (DM) status, and HTN status as covariates. To assess the robustness of the adjusted HD-versus-KTR comparisons, sensitivity analyses were performed by repeating the GEE models after excluding participants with DM and by replacing binary HTN status with HTN grade (0–3).

Additional exploratory analyses were performed to evaluate the association of primary renal disease category and current pharmacologic treatment with OCT/OCTA parameters in each of the renal cohorts. Because several individual etiologies were rare, primary renal diseases were grouped into broader categories: unknown/unspecified, glomerular/immune, congenital/hereditary, vascular/metabolic, and tubulointerstitial/other.

Medication-related analyses were performed separately within the HD and KTR groups. Medication exposure was analyzed according to major therapeutic classes, including statins, antihypertensive medication classes in each group, and immunosuppressive therapy in KTR. These exploratory models were fitted using Gaussian GEE with an exchangeable working correlation, clustered by patient and including both eyes. Models were adjusted for age, sex, BMI, DM status and HTN status. Because of small subgroup sizes, multiple exploratory comparisons, and the absence of correction for multiple testing, these analyses were interpreted as hypothesis-generating. A two-tailed *p*-value < 0.05 was considered statistically significant.

## 3. Results

### 3.1. Descriptive Data

Baseline demographic, retinal, and systemic parameters are summarized in Table 1. KTR were significantly younger than HD patients (48.19 ± 10.67 vs. 57.97 ± 14.29 years; *p* = 0.003); whereas, BMI was comparable between groups (*p* = 0.989). BVCA did not differ significantly between groups in either eye (RE, *p* = 0.065; LE, *p* = 0.110).

Mean dialysis vintage in the HD group was 77.5 ± 76.1 months. In the KTR group, the mean time since transplantation was 101.2 ± 76.0 months, and 28 patients had undergone HD prior to graft receipt, with a mean duration of 25.4 ± 36 months.

The distribution of underlying kidney disease did not differ significantly between HD and KTR overall (*p* = 0.366). Unknown or unspecified kidney disease was the most frequent category in both groups, affecting eight HD patients (27.6%) and 17 KTR patients (35.4%). This was followed by autosomal dominant polycystic kidney disease, recorded in three HD patients (10.3%) and eight KTR patients (16.7%), and chronic glomerulonephritis, recorded in three HD patients (10.3%) and five KTR patients (10.4%). Hypertensive nephrosclerosis and diabetic kidney disease were numerically more frequent in HD than in KTR, each being present in four HD patients (13.8%) versus one KTR patient (2.1%), but these differences did not reach statistical significance.

All KTR were receiving maintenance immunosuppressive therapy. Mycophenolate mofetil was the most commonly used antiproliferative agent (47/48, 97.9%), followed by tacrolimus as the predominant calcineurin inhibitor (37/48, 77.1%), with ciclosporin used in a minority of patients (8/48, 16.7%); one patient was receiving azathioprine (1/48, 2.1%). Corticosteroids (prednisone) were part of the regimen in 42 patients (87.5%). Three patients were not on a calcineurin inhibitor at the time of assessment, which may reflect protocol-driven minimization or withdrawal (Table 1).

The overall prevalence of HTN did not differ significantly between groups (*p* = 0.087), although the distribution of HTN grade differed (*p* = 0.001). Most cardiovascular medications were similarly distributed between the two renal cohorts. Beta blockers were used by 12 HD patients (41.4%) and 24 KTR patients (50.0%), statins by eight HD patients (27.6%) and 16 KTR patients (33.3%), angiotensin-converting enzyme inhibitors (ACEI) by one HD patient (3.4%) and four KTR patients (8.3%), angiotensin II receptor blockers (ARB) by six HD patients (20.7%) and four KTR patients (8.3%), calcium channel blockers (CCB) by 10 HD patients (34.5%) and 17 KTR patients (35.4%), and central antihypertensive agents by four HD patients (13.8%) and nine KTR patients (18.8%). Diuretic use was significantly more frequent in HD than in KTR, being recorded in 12 HD patients (41.4%) versus five KTR patients (10.4%; *p* = 0.004).

The prevalence of DM was similar between groups (*p* = 1.0, Table 1). Six KTR patients (33.3%) versus eight HD patients (27.6%) were using statins (*p* = 0.8).

On OCTA, SVPD, DVPD, and CC density did not differ significantly among groups (all *p* > 0.05). FAZ area was likewise comparable between groups bilaterally (RE, *p* = 0.245; LE, *p* = 0.462).

On structural OCT analysis, GCL+, GCL++ and CMT did not differ significantly between HD and KTR in either eye (all *p* > 0.05). SCT was significantly lower in HD patients than in KTR in the RE (189.44 ± 29.61 vs. 209.83 ± 43.41 µm; *p* = 0.019); whereas, the LE difference did not reach statistical significance (Table 1).

Regarding mineral metabolism, HD patients had significantly lower corrected calcium and higher phosphate, magnesium, and PTH than KTR (all *p* < 0.05). Serum 25-hydroxyvitamin D levels, available in a subset of participants (HD, *n* = 10; KTR, *n* = 10), did not differ significantly among groups (*p* = 0.789); however, this comparison is limited by the small sample size. Urea, creatinine, and eGFR were reported for KTR only, as these values are not directly interpretable in maintenance HD.

### 3.2. GEE-Adjusted and Sensitivity Analyses

After adjustment, no significant between-group difference was observed for FAZ area (adjusted B = −16.90 µm^2^; 95% CI −58.08 to 24.27; *p* = 0.421), GCL+ (B = −0.71 µm; *p* = 0.666), GCL++ (B = −3.42 µm; *p* = 0.171), CMT (B = +6.44 µm; *p* = 0.189), SVPD (B = +0.82%; *p* = 0.462), DVPD (B = +0.91%; *p* = 0.431), or CC density (B = −0.36; *p* = 0.806). SCT remained significantly higher in KTR compared to HD (adjusted B = +22.62 µm; 95% CI 4.21 to 41.04; *p* = 0.016), consistent with the unadjusted per-eye finding of a between-group difference in SCT (Table 2).

Several sensitivity analyses were performed to assess the robustness of the adjusted two-cohort GEE results (Table 3). After excluding participants with DM, no significant differences between KTR and HD were observed for FAZ, GCL+, GCL++, CMT, SVPD, DVPD, or CC density, while SCT remained significantly higher in KTR than in HD (adjusted B = +21.62 µm, *p* = 0.044). Similar results were obtained when HTN was modeled as an ordinal grade (0–3) instead of a binary covariate: the principal retinal microvascular and inner retinal structural parameters remained comparable between the two renal cohorts; whereas, SCT remained higher in KTR compared with HD (adjusted B = +27.37 µm, *p* = 0.009).

Exploratory analyses of renal disease etiology and pharmacologic treatment were additionally performed (Supplementary Tables S1 and S2). In stratified analyses, primary renal disease category showed exploratory associations with several OCT/OCTA parameters within both renal cohorts. In the HD group, compared with patients with unknown or unspecified renal disease etiology, glomerular/immune disease was associated with lower CMT, while vascular/metabolic disease was associated with lower SCT. Glomerular/immune disease was also associated with lower SVPD. In the KTR group, glomerular/immune disease was associated with higher FAZ and lower SVPD compared with unknown or unspecified etiology, while tubulointerstitial/other disease was associated with higher CMT and higher GCL+. Congenital/hereditary disease was also associated with higher GCL+ in KTR (Supplementary Table S1).

Medication-related analyses were also performed separately within each renal cohort. Statin use was not significantly associated with OCT/OCTA parameters in either HD or KTR. In the HD group, CCB use was associated with lower GCL++ and lower SCT, while central antihypertensive use was associated with lower GCL++. In the KTR group, ACEI/ARB use was associated with lower GCL+ and lower SCT; any antihypertensive medication use was associated with lower GCL+ and GCL++; beta blocker use was associated with lower GCL++; and CCB use was associated with higher FAZ. In the KTR subgroup, tacrolimus-treated patients had lower GCL+ than cyclosporine A-treated patients, while no significant differences were observed for the other OCT/OCTA parameters (Supplementary Table S2).

### 3.3. Correlation Analysis

Significant correlations within each group are summarized in Table 4. In HD, but not in KT, age was inversely associated with BCVA; in HD patients, this extended to the inner retinal layers (GCL+ and GCL++); whereas, no such age-related structural associations were observed in the KTR. In HD patients, associations with mineral metabolism markers were limited to single-eye findings: SCT correlated positively with PTH, SVPD inversely with corrected calcium, and FAZ area inversely with magnesium (Table 4).

In KTR, prior dialysis duration was inversely correlated with BCVA in both eyes but was not associated with any retinal parameter. GCL+ correlated negatively with PTH bilaterally and positively with magnesium in the LE. Cumulative GC dose was inversely associated with CC density in the RE. Time since transplantation was not correlated with any retinal parameter.

Correlations between HTN grade and retinal parameters were also explored within each group. In HD patients, HTN grade was positively correlated with FAZ area in the LE (ρ = 0.647, *p* < 0.001) and inversely correlated with DVPD bilaterally (RE: ρ = −0.430, *p* = 0.025; LE: ρ = −0.377, *p* = 0.044); and in KTR, HTN grade was positively correlated with SCT bilaterally (RE: ρ = 0.348, *p* = 0.015; LE: ρ = 0.311, *p* = 0.031).

### 3.4. Regression Analysis

Bivariate linear regression analyses were performed within each group to explore independent associations between systemic parameters and ocular measurements. Significant results are depicted in Table 5.

In the HD group, age remained independently associated with thinner GCL+ and GCL++ parameters in both eyes. Corrected calcium was associated with lower SVPD in the LE, and HTN grade was negatively associated with DVPD in the LE. In the KTR group, most biochemical associations were attenuated after adjustment; whereas, HTN grade remained independently associated with increased SCT in both eyes (Table 5).

**Figure 1:**
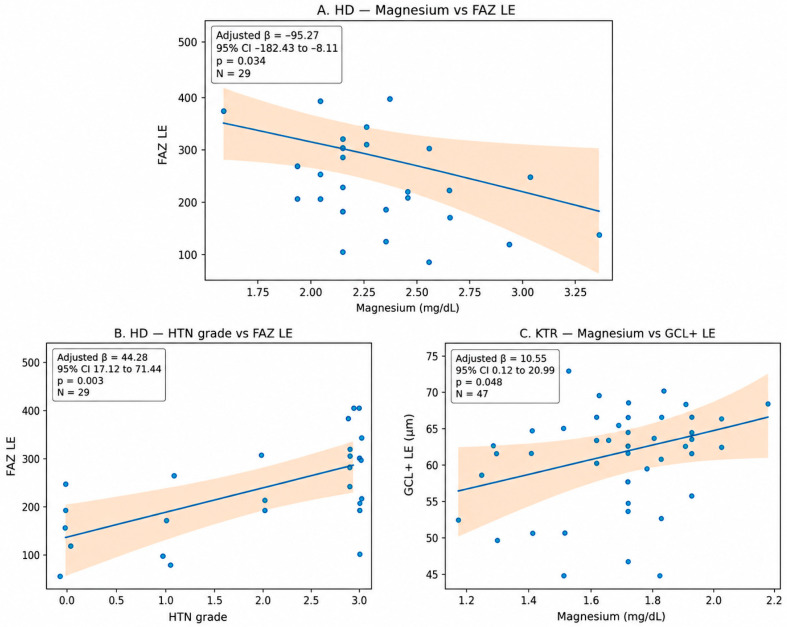
Representative systemic–ocular associations across study groups. (**A**) Association between serum magnesium and FAZ LE in the HD group, adjusted for hypertension grade, age, sex, and diabetes status. (**B**) Association between HTN grade and FAZ LE in the HD group, adjusted for serum magnesium, age, sex, and DM status. (**C**) Association between serum magnesium and ganglion cell layer plus inner plexiform layer thickness in the left eye (GCL+ LE) in KTR, adjusted for PTH, age, sex, DMstatus, and HTN grade. Lines represent adjusted predicted regression lines, and shaded areas represent 95% confidence intervals. Dots represent individual observations. HD, hemodialysis; KTR, kidney transplant recipients; PTH, parathyroid hormone. Abbreviations: FAZ, foveal avascular zone; GCL+, ganglion cell layer + inner plexiform layer; HD, hemodialysis; KTR, kidney transplant recipients; HTN, hypertension (grade 0–3); LE, left eye; DM, diabetes mellitus.

Additional multiple regression models were fitted for parameters with more than one significant correlate (Table 6). In the HD group, both magnesium and HTN grade remained independent predictors of the LE FAZ (adjusted R^2^ = 0.382, *p* = 0.001, Figure 1). In the KTR group, the multiple regression model for GCL+ LE was not significant overall (adjusted R^2^ = 0.061, *p* = 0.203); however, magnesium remained positively associated with GCL+ LE; whereas, PTH did not (Table 6, Figure 1).

## 4. Discussion

This cross-sectional study compared retinal structural and microvascular parameters between patients with CKD treated by maintenance HD and those treated by kidney transplantation. Across per-eye, adjusted, and sensitivity analyses, most retinal microvascular parameters (SVPD, DVPD, CCD, and FAZ area) and inner-retinal structural parameters (GCL+, GCL++, and CMT) were comparable between the two cohorts, with the exception of SCT, which was lower in HD patients than in KTR in adjusted GEE models. SCT also remained significantly lower after excluding diabetic patients and after adjustment for HTN grade.

Within each group, serum magnesium was the metabolic marker most consistently associated with retinal parameters—inversely with FAZ area in HD patients and positively with GCL+ thickness in KTR—and HTN grade was the clinical variable most frequently associated with retinal parameters, remaining independently associated with FAZ area in HD patients and with SCT in KTR. Corrected serum calcium was also inversely associated with SVPD in HD patients, persisting after adjustment. Neither dialysis vintage in the HD group nor time since transplantation in KTR was associated with any retinal parameter.

### 4.1. Inner-Retinal and Microvascular Parameters

Neither the microvascular metrics (SVPD, DVPD, CCD, and FAZ area) nor the inner-retinal and macular thickness measures (GCL+, GCL++, and CMT) differed significantly between the two cohorts, either in unadjusted per-eye comparisons or in the confirmatory GEE model adjusted for age, sex, BMI, DM, and HTN and this pattern was preserved after excluding participants with DM and after adjustment for HTN grade.

First, these parameters may reach a broadly similar state in stable maintenance HD and in long-standing KTR, such that the treatment modality itself is not strongly associated with them once demographic and cardiometabolic covariates are accounted for. This would be consistent with reports that CKD-associated retinal structural and microvascular alterations relate more closely to cumulative disease severity and systemic microvascular burden than to any single treatment status [9,20,21,22,23,24,25,26,27,28,29,30]. Second, because the available sample was powered to detect only moderate-to-large HD-versus-KTR differences, smaller between-cohort differences may have escaped detection, and the absence of a statistically significant difference.

### 4.2. Subfoveal Choroidal Thickness

SCT was the main parameter distinguishing the two cohorts, being lower in HD patients and higher in KTR across the adjusted and sensitivity analyses. This pattern, although partly driven by the right eye, is directionally consistent with the report of Farrah et al. [31], who described choroidal thinning in CKD that was reversed following transplantation. The choroid is mainly regulated by autonomic innervation and by systemic vasoactive factors (e.g., catecholamines, angiotensin II, and endothelin-1) rather than by intrinsic autoregulation and may therefore be particularly susceptible to the hemodynamic fluctuations and volume shifts associated with HD [11,31,32]. The selective persistence of the SCT difference across adjusted and sensitivity analyses is compatible with this susceptibility, although a cross-sectional design cannot confirm it. In KTR, HTN grade was positively correlated with SCT bilaterally and remained an independent predictor in regression analysis; this association is exploratory and its mechanism is uncertain, but it suggests that choroidal measures in this group may be influenced by cardiovascular comorbidity. Overall, these observations should be interpreted as an association between treatment modality and SCT rather than as evidence of a causal or reversible effect of transplantation.

### 4.3. Hypertension and Retinal Findings

Beyond its inclusion as a covariate in the confirmatory GEE model, HTN grade was the clinical variable most frequently associated with retinal parameters in the within-group analyses, and several of these associations persisted in the adjusted regression models. In HD patients, higher HTN grade was associated with a larger FAZ area in the LE and with lower DVPD, and it remained an independent predictor of FAZ area together with magnesium. In KTR, HTN grade was positively and independently associated with SCT in both eyes. These findings are consistent with the established role of systemic HTN in retinal microvascular and choroidal remodeling and suggest that, in this cohort, blood pressure burden contributed to retinal changes alongside CKD-related factors [33,34]. This interpretation is also compatible with the exploratory medication-class analyses, in which several antihypertensive classes were associated with retinal parameters—including lower GCL++ and lower SCT among CCB users in HD, and lower GCL+ and lower SCT among ACEI/ARB users in KTR—although, because antihypertensive use is closely linked to HTN severity, these medication associations cannot be separated from the underlying blood-pressure burden.

### 4.4. Mineral Metabolism and Retinal Structure

CKD-MBD is characterized by disturbances in calcium–phosphate homeostasis, secondary hyperparathyroidism, and progressive vascular calcification [35]. Serum PTH correlated negatively with GCL+ thickness bilaterally in KTR. Elevated PTH levels have been linked to endothelial dysfunction, vascular stiffness, and microvascular injury [36], mechanisms that may plausibly extend to the retinal circulation. However, PTH did not reach significance in linear regression models, suggesting that this relationship may be non-linear, confounded by other variables, or influenced by skewed distribution of PTH in our cohort. Corrected serum calcium showed associations of small magnitude confined to single analyses (inversely with SVPD in the LE in HD patients); their biological significance may reflect the role of calcium in vascular and neuronal homeostasis [36,37] but should be regarded as exploratory.

Serum magnesium emerged as the most consistent metabolic correlate of retinal structure across both CKD groups. In HD patients, lower magnesium was associated with larger FAZ area, and magnesium remained an independent predictor in a multiple regression model that also included HTN grade, the strongest regression finding in this study. In KTR, higher magnesium was associated with greater GCL+ thickness and remained an independent predictor when entered together with PTH and demographic covariates, although the overall multivariable model for GCL+ was not statistically significant. Magnesium plays a recognized role in endothelial function, vascular tone regulation, and inhibition of vascular calcification [38], and its potential role in retinal neuroprotection warrants prospective investigation. Except for the FAZ model in HD patients, the coefficients of determination for these metabolic associations were low, and several biochemical associations obtained in unadjusted analysis were attenuated after adjustment. Each systemic parameter therefore accounted for only a small fraction of the variance in the corresponding retinal measure.

### 4.5. Immunosuppression, Dialysis Burden and Visual Function

Although the inverse association between cumulative GC exposure and CC density in KTR was unilateral and did not reach statistical significance in regression analysis, it is of interest given the recognized association between corticosteroid use and choroidal vascular changes, most notably in central serous chorioretinopathy [39,40]. Larger studies are warranted to examine whether long-term immunosuppressive GC exposure affects CC perfusion in KTR.

In the exploratory medication-class analysis within the KTR subgroup, tacrolimus-treated patients had lower GCL+ thickness than ciclosporin-treated patients; whereas, the other OCT/OCTA parameters did not differ significantly between the two calcineurin-inhibitor groups. This observation should be interpreted with particular caution, as the ciclosporin subgroup was small. Nonetheless, it is of mechanistic interest: both calcineurin inhibitors have been associated with neurotoxicity and with vascular and endothelial effects in experimental and clinical studies [41], and calcineurin signaling has been implicated in retinal ganglion cell integrity in preclinical models [42]. Whether these pathways underlie a differential association of specific calcineurin inhibitors with inner-retinal thickness in KTR cannot be determined from the present cross-sectional data and requires dedicated, adequately powered study.

Duration of prior HD was negatively associated with BCVA bilaterally in KTR, without corresponding structural OCT correlates. Chronic dialysis exposure has been associated with microvascular dysfunction, hemodynamic instability, and repetitive ischemia–reperfusion events [20]. While the cumulative systemic burden of pre-transplant dialysis exposure may affect visual function independently of measurable OCT changes, it is also possible that the observed functional impairment reflects mechanisms not captured by the structural parameters assessed in this study.

#### CKD Etiology and Retinal Findings

The distribution of primary renal disease was comparable between the two cohorts with unknown or unspecified etiology being the most frequent category in both groups. In exploratory stratified analyses, certain etiologic categories were associated with individual OCT/OCTA parameters—for example, glomerular/immune disease with lower SVPD in both cohorts, and vascular/metabolic disease with lower SCT in HD patients—a pattern broadly compatible with reports that immune-mediated and vascular renal disease may share microvascular features with the retinal circulation [43,44]. However, the etiologic subgroups were small and unevenly distributed, a large proportion of patients had unknown or unspecified disease, and multiple comparisons were performed without correction.

### 4.6. Strengths and Limitations

To our knowledge, this is the first study to examine the association between mineral metabolism parameters and retinal structural measurements in HD patients and KTR. The study integrates structural OCT and OCTA microvascular analysis in both HD patients and KTR, allowing comparison between ongoing renal replacement and post-transplant states. Manual FAZ quantification by a single masked examiner ensured consistent measurement across participants, and the confirmatory GEE model—adjusted for age, sex, BMI, DM, and HTN and accounting for inter-eye correlation—supported the robustness of the principal between-group differences.

Several limitations should be acknowledged. The cross-sectional design precludes causal inference. The analyzed dataset compared two renal replacement cohorts and did not include a non-CKD reference group, so it cannot establish whether either cohort differs from individuals without CKD. Inner-retinal and macular thickness parameters are age-dependent, and the younger age of the KTR group is a potential confounder for structural comparisons; the between-cohort structural parameters were nonetheless comparable after adjustment for age in the GEE model. As shown by the sensitivity analysis, only medium-to-large effects were reliably detectable, and small-magnitude associations may have escaped detection. Vitamin D measurements were available only in a limited subset, precluding robust analysis for this variable. Finally, the low R^2^ values in the regression models indicate that the examined systemic parameters explain only a small proportion of retinal variance, and unmeasured confounders likely play a substantial role.

## 5. Conclusions

In this cross-sectional comparison of two renal replacement cohorts, most retinal microvascular and inner-retinal structural parameters were similar in HD patients and KTR; whereas, SCT was consistently higher in KTR, persisting after adjusted and sensitivity analyses. This pattern is directionally consistent with prior reports of choroidal thinning in CKD and its partial recovery after transplantation, although a cross-sectional design cannot establish causality or reversibility. Among the metabolic markers examined, serum magnesium was the most consistent correlate of retinal parameters and merits prospective evaluation as a potentially modifiable metabolic correlate of retinal integrity. HTN grade was the clinical variable most consistently associated with retinal parameters, remaining independently related to FAZ area in HD patients and to SCT in KTR, underscoring the contribution of blood-pressure burden to the retinal changes observed in this population. The planned one-year prospective follow-up of this cohort is intended to determine whether the observed retinal parameters are progressive, modifiable, or predictive of clinical outcomes.

## Figures and Tables

**Table 1:** Between-group comparison of demographic, retinal, mineral metabolism, and diabetes parameters.

Parameter	HD (*n* = 29)	KTR (*n* = 48)	*p*
Age (years)	57.97 ± 14.29 ^a^	48.19 ± 10.67 ^a^	0.003
BMI (kg/m^2^)	27.62 ± 4.24	27.61 ± 4.87	0.989
BCVA RE	0.79 ± 0.31	0.91 ± 0.15	0.065
BCVA LE	0.83 ± 0.21	0.90 ± 0.20	0.110
SVPD RE (%)	23.98 ± 10.34	23.57 ± 6.48	0.852
SVPD LE (%)	22.91 ± 5.25	23.73 ± 5.80	0.528
DVPD RE (%)	24.45 ± 10.56	24.34 ± 6.37	0.962
DVPD LE (%)	23.45 ± 5.16	24.31 ± 5.69	0.495
CCD RE	20.99 ± 11.79	19.76 ± 7.97	0.631
CCD LE	19.50 ± 6.05	19.07 ± 9.03	0.802
FAZ RE (µm^2^)	263.83 ± 131.14	229.40 ± 101.95	0.245
FAZ LE (µm^2^)	244.11 ± 99.66	227.73 ± 83.69	0.462
GCL+ RE (µm)	62.81 ± 6.42	61.08 ± 7.47	0.295
GCL+ LE (µm)	59.97 ± 8.94	61.21 ± 7.41	0.532
GCL++ RE (µm)	107.22 ± 12.43	101.98 ± 12.08	0.082
GCL++ LE (µm)	103.48 ± 12.51	104.73 ± 12.08	0.670
CMT RE (µm)	232.67 ± 14.50	237.77 ± 18.30	0.189
CMT LE (µm)	237.07 ± 31.09	241.06 ± 19.41	0.537
SCT RE (µm)	189.44 ± 29.61 ^a^	209.83 ± 43.41 ^a^	0.019
SCT LE (µm)	196.17 ± 36.50	211.44 ± 41.40	0.096
Corrected calcium (mg/dL)	8.61 ± 0.73 ^a^	9.07 ± 0.57 ^a^	0.006
Magnesium (mg/dL)	2.26 ± 0.38 ^a^	1.67 ± 0.22 ^a^	<0.001
Phosphate (mg/dL)	5.08 ± 1.31 ^a^	2.91 ± 0.62 ^a^	<0.001
PTH (pg/mL)	497.26 ± 536.84 ^a^	120.37 ± 196.45 ^a^	<0.001
25-OH vitamin D (ng/mL)	29.50 ± 15.58	26.58 ± 11.37	0.638
Urea (mg/dL)	—	55.52 ± 21.47	-
Creatinine (mg/dL)	—	1.51 ± 0.65	-
eGFR (mL/min/1.73 m^2^)	—	57.52 ± 21.02	-
HTN, *n* (%)	24 (82.8)	31 (64.6)	0.087
HTN grade [0/1/2/3], *n*	5/5/4/15 ^a^	17/20/5/6 ^a^	0.001
DM, *n* (%) [T1/T2]	4 (13.8) [0/4]	7 (14.6) [1/6]	1

Urea, creatinine, and eGFR are not reported for the HD group because, in maintenance HD, these values reflect dialysis timing and residual clearance rather than intrinsic renal function and are not directly comparable with the non-dialysis groups. 25-OH vitamin D was available in a subset of participants (HD, *n* = 10; KTR, *n* = 10). ^a^ Indicates a statistically significant difference between the HD and KTR groups (*p* < 0.05).Abbreviations: BCVA, best-corrected visual acuity; RE, right eye; LE, left eye; SVPD, superficial vascular plexus density; DM, diabetes mellitus; DVPD, deep vascular plexus density; CCD, choriocapillaris density; FAZ, foveal avascular zone; GCL+, ganglion cell layer plus inner plexiform layer; GCL++, ganglion cell complex; CMT, central macular thickness; SCT, subfoveal choroidal thickness; HD, hemodialysis; KTR, kidney transplant recipients; PTH, parathyroid hormone; BMI, body mass index; HTN, hypertension (grade coded 0 [absent] to 3); T1, type 1 diabetes; T2, type 2 diabetes.

**Table 2:** Confirmatory GEE analysis of between-group differences in retinal parameters, adjusted for age, sex, BMI, DM, and HTN (77 patients, 154 eyes).

Outcome	Adjusted B	95% CI	*p*
FAZ (µm^2^)	−16.90	−58.08 to 24.27	0.421
GCL+ (µm)	−0.71	−3.93 to 2.51	0.666
GCL++ (µm)	−3.42	−8.32 to 1.48	0.171
CMT (µm)	+6.44	−3.17 to 16.04	0.189
SCT (µm)	+22.62	4.21 to 41.04	0.016
SVPD (%)	+0.82	−1.37 to 3.01	0.462
DVPD (%)	+0.91	−1.36 to 3.19	0.431
CC density	−0.36	−3.24 to 2.52	0.806

B values are adjusted regression coefficients (mean difference) from a Gaussian GEE model with an exchangeable working correlation, clustered by patient, including both eyes and adjusted for age, sex, BMI, DM, and HTN. Abbreviations: GEE, generalized estimating equation; FAZ, foveal avascular zone; GCL+, ganglion cell layer plus inner plexiform layer; GCL++, ganglion cell complex; CMT, central macular thickness; SCT, subfoveal choroidal thickness; BMI, body mass index; CI, confidence interval.

**Table 3:** Sensitivity analyses of the confirmatory GEE model for the main retinal outcomes.

Outcome	Sensitivity Analysis	Contrast	Adjusted B	95% CI	*p*
FAZ (µm^2^)	Non-diabetic only	KTR vs. HD	−13.88	−55.10 to 27.35	0.509
	HTN grade adjustment		−3.25	−41.41 to 34.90	0.867
GCL+ (µm)	Non-diabetic only		−0.70	−4.03 to 2.63	0.681
	HTN grade adjustment		−0.33	−3.96 to 3.30	0.860
GCL++ (µm)	Non-diabetic only		−4.09	−9.39 to 1.21	0.131
	HTN grade adjustment		−3.33	−9.00 to 2.34	0.250
CMT (µm)	Non-diabetic only		+5.14	−4.55 to 14.83	0.298
	HTN grade adjustment		+7.43	−3.49 to 18.35	0.182
SCT (µm)	Non-diabetic only		+21.62	0.63 to 42.61	0.044
	HTN grade adjustment		+27.37	6.92 to 47.82	0.009
SVPD (%)	Non-diabetic only		+0.02	−2.37 to 2.41	0.985
	HTN grade adjustment		+0.70	−1.60 to 3.01	0.549
DVPD (%)	Non-diabetic only		+0.05	−2.46 to 2.55	0.971
	HTN grade adjustment		+0.75	−1.58 to 3.08	0.529
CC density	Non-diabetic only		−0.86	−3.99 to 2.26	0.589
	HTN grade adjustment		−0.88	−3.62 to 1.87	0.531

All models are Gaussian GEE models with an exchangeable working correlation, clustered by patient and including both eyes. The contrast shown is KTR versus HD; positive B values indicate higher values in KTR compared with HD. The non-diabetic analysis excluded participants with DM and was adjusted for age, sex, BMI, and HTN status. The HTN grade model included all HD and KTR participants and was adjusted for age, sex, BMI, DM, and HTN grade (0–3) instead of binary HTN status. Sample sizes: non-diabetic only, 66 patients/132 eyes; HTN grade model, 77 patients/154 eyes. Abbreviations: GEE, generalized estimating equation; FAZ, foveal avascular zone; GCL+, ganglion cell layer plus inner plexiform layer; GCL++, ganglion cell complex; CMT, central macular thickness; SCT, subfoveal choroidal thickness; BMI, body mass index; HD, hemodialysis; KTR, kidney transplant recipients; CI, confidence interval; HTN, hypertension (grade 0–3).

**Table 4:** Significant correlations between retinal and demographic and systemic parameters by study group.

Group	Retinal/Ocular Parameter (Variable 1)	Systemic/Clinical Parameter (Variable 2)	r/ρ	*p*
HD	BCVA RE	Age	−0.389	0.037
	BCVA LE	Age	−0.679	<0.001
	GCL+ RE	Age	−0.479	0.012
	GCL+ LE	Age	−0.389	0.037
	GCL++ RE	Age	−0.435	0.023
	GCL++ LE	Age	−0.421	0.023
	SVPD LE	Corrected calcium	−0.404	0.030
	FAZ LE	Magnesium	−0.414	0.026
		HTN grade	0.647	<0.001
	SCT RE	PTH	0.456	0.017
	DVPD RE	HTN grade	−0.430	0.025
	DVPD LE	HTN grade	−0.377	0.044
KTR	mRNFL LE	Urea	0.310	0.032
	GCL+ RE	PTH	−0.383	0.007
	GCL+ LE	PTH	−0.376	0.009
		Magnesium	0.321	0.028
	CC RE	Cumulative GC dose	−0.310	0.046
	BCVA RE	Prior HD duration	−0.384	0.007
	BCVA LE	Prior HD duration	−0.299	0.039
	SCT RE	HTN grade	0.348	0.015
	SCT LE	HTN grade	0.311	0.031

Only statistically significant correlations are listed. Abbreviations: BCVA, best-corrected visual acuity; FAZ, foveal avascular zone; GCL+, ganglion cell layer plus inner plexiform layer; GCL++, ganglion cell complex; SVPD, superficial vascular plexus density; CC, choriocapillaris; HD, hemodialysis; PTH, parathyroid hormone; KTR, kidney transplant recipients; mRNFL, macular retinal nerve fiber layer; RE, right eye; LE, left eye; SCT, subfoveal choroidal thickness; GC, glucocorticoid; HTN, hypertension (grade 0–3); DVPD, deep vascular plexus density.

**Table 5:** Adjusted linear regression models for significant within-group correlations.

Group	Dependent Variable	Predictor	Adjusted β	Adjusted 95% CI	Adjusted *p*
HD	GCL+ RE	Age	−0.20	−0.38 to −0.02	0.030
	GCL+ LE		−0.24	−0.48 to −0.00	0.050
	GCL++ RE		−0.39	−0.73 to −0.04	0.029
	GCL++ LE		−0.38	−0.71 to −0.05	0.024
	SVPD LE	Corrected calcium	−2.98	−5.21 to −0.75	0.011
	SCT RE	PTH	0.020	−0.004 to 0.043	0.091
	DVPD RE	HTN grade	−2.38	−6.33 to 1.57	0.225
	DVPD LE		−2.03	−3.52 to −0.54	0.010
KTR	mRNFL LE	Urea	0.10	−0.03 to 0.24	0.132
	GCL+ RE	PTH	−0.008	−0.019 to 0.003	0.164
	SCT RE	HTN grade	15.62	2.77 to 28.47	0.018
	SCT LE		15.85	3.36 to 28.34	0.014

Values are regression coefficients β with 95% confidence intervals. Unadjusted models included only the predictor of interest. Adjusted models were fitted separately within each study group. Age-related models were adjusted for sex. Models including biochemical or clinical predictors were adjusted for age, sex, diabetes status, and HTN grade, except when HTN grade was the predictor of interest, in which case adjustment included age, sex, and diabetes status. Abbreviations: CMT, central macular thickness; mRNFL, macular retinal nerve fiber layer; GCL+, ganglion cell layer plus inner plexiform layer; GCL++, ganglion cell complex; SVPD, superficial vascular plexus density; DVPD, deep vascular plexus density; SCT, subfoveal choroidal thickness; PTH, parathyroid hormone; RE, right eye; LE, left eye; HTN, hypertension (grade 0–3).

**Table 6:** Multiple liniar regression models for selected ocular parameters.

Group	Dependent Variable	Predictor	β	95% CI	*p*-Value
HD	FAZ LE	Magnesium	−95.27	−182.43 to −8.11	0.034
		HTN grade	44.28	17.12 to 71.44	0.003
		Age	−0.09	−2.63 to 2.45	0.943
		Male sex	−4.76	−71.54 to 62.01	0.884
		DM status	50.09	−51.61 to 151.78	0.319
KTR	GCL+ LE	PTH	−0.006	−0.017 to 0.005	0.275
		Magnesium	10.55	0.12 to 20.99	0.048
		Age	−0.07	−0.30 to 0.15	0.519
		Male sex	−0.94	−5.56 to 3.68	0.683
		DM status	−4.29	−10.80 to 2.22	0.191
		HTN grade	1.10	−1.37 to 3.56	0.375

Abbreviations: HD, hemodialysis; KTR, kidney transplant recipients; FAZ, foveal avascular zone; GCL+, ganglion cell layer plus inner plexiform layer; GCL++, ganglion cell complex; PTH, parathyroid hormone; RE, right eye; LE, left eye; HTN, hypertension (grade 0–3); DM, diabetes mellitus;.

## Data Availability

The original contributions presented in this study are included in the article/Supplementary Materials. Further inquiries can be directed to the corresponding author.

## Supplementary Materials

The following supporting information can be downloaded at: https://www.mdpi.com/article/10.3390/medsci14030381/s1, Table S1: Stratified exploratory associations of primary renal disease with OCT/OCTA parameters; Table S2: Stratified exploratory associations of medication use (users vs. non-users) and OCT/OCTA parameters, Supplementary Figure S1. Representative OCTA acquisition from a hemodialysis patient obtained using swept-source OCT angiography (DRI OCT Triton, Topcon).

## Abbreviations

The following abbreviations are used in this manuscript:

Abbreviation	Full term
ACEI	Angiotensin-converting enzyme inhibitors
ARB	Angiotensin II receptor blockers
ANOVA	One-way analysis of variance
BCVA	Best corrected visual acuity
BMI	Body mass index
CC	Choriocapillaris
CCB	Calcium channel blockers
CCD	Choriocapillaris density
CI	Confidence interval
CKD	Chronic kidney disease
CKD-MBD	CKD-related mineral and bone disorder
CMT	Central macular thickness
DM	Diabetes mellitus
DVP	Deep vascular plexus
DVPD	Deep vascular plexus density
ECLIA	Electrochemiluminescence immunoassay
eGFR	Estimated glomerular filtration rate
FAZ	Foveal avascular zone
GC	Glucocorticoid
GCL+	Ganglion cell layer plus inner plexiform layer
GCL++	Ganglion cell complex
GEE	Generalized estimating equation
HD	Hemodialysis
HTN	Hypertension
ICC	Intraclass correlation coefficient
ILM	Internal limiting membrane
INL	Inner nuclear layer
IOP	Intraocular pressure
iPTH	Intact parathyroid hormone
IPL	Inner plexiform layer
KT	Kidney transplant
KTR	Kidney transplant recipients
LE	Left eye
mRNFL	Macular retinal nerve fiber layer
OCT	Optical coherence tomography
OCTA	Optical coherence tomography angiography
PD	Perfusion density
PTH	Parathyroid hormone
RE	Right eye
RNFL	Retinal nerve fiber layer
SCT	Subfoveal choroidal thickness
SD	Standard deviation
SVP	Superficial vascular plexus
SVPD	Superficial vascular plexus density
