# Supplementary material for: Retinal Structural and Microvascular Findings on OCT and OCTA in Hemodialysis and Kidney Transplant Recipients and Their Association with Mineral Metabolism Parameters
Source: Med Sci (Basel). 2026 Jul 9;14(3):381. doi: 10.3390/medsci14030381 (PMC13413533; doi:10.3390/medsci14030381)
Supplement: Supplementary file 1 [file medsci-14-00381-s001.zip › medsci-4417732-supplementary.pdf]

Additional exploratory analyses were performed to evaluate the association of primary renal disease category (Table S1A) and current pharmacologic treatment (Table S1B) with OCT/OCTA parameters separately within the HD and KTR cohorts. Primary renal diseases were grouped into broader categories: unknown/unspecified, glomerular/immune, congenital/hereditary, vascular/metabolic, and tubulointerstitial/other. Medication exposure was analyzed according to major therapeutic classes, including statins, antihypertensive medication classes, and diuretics. Models were fitted using Gaussian generalized estimating equations with an exchangeable working correlation, clustered by patient and including both eyes, and were adjusted for age, sex, BMI, DM status, and HTN status when covariates had within-cohort variation. Because of small subgroup sizes, multiple exploratory comparisons, and the absence of correction for multiple testing, these analyses were interpreted as hypothesis-generating.

Table S1. Stratified exploratory associations of primary renal disease with OCT/OCTA parameters

Cohort	Contrast	Outcome	B	95% CI	p	Global p
HD N=27 patients (52 eyes)	Congenital/hereditary vs unknown/unspecified (N=5 vs. N=8)	CMT (µm)	1.86	-19.87 to 23.59	0.867	0.033
		GCL++ (µm)	-2.87	-12.71 to 6.97	0.567	0.020
		SCT (µm)	-15.44	-52.75 to 21.87	0.417	<0.001
		SVPD (%)	2.63	-6.50 to 11.75	0.573	0.059
	Glomerular/immune vs unknown/unspecified (N=6 vs. N=8)	CMT (µm)	-18.14	-33.72 to -2.56	0.023	0.033
		GCL++ (µm)	3.16	-4.41 to 10.74	0.413	0.020
		SCT (µm)	-9.35	-41.88 to 23.18	0.573	<0.001
		SVPD (%)	-5.68	-10.80 to -0.57	0.029	0.059
	Vascular/metabolic vs unknown/unspecified (N=8 vs. N=8)	CMT (µm)	2.58	-21.81 to 26.97	0.836	0.033
		GCL++ (µm)	-8.93	-19.23 to 1.37	0.089	0.020
		SCT (µm)	-60.75	-98.85 to -22.66	0.002	<0.001
		SVPD (%)	-3.19	-13.01 to 6.62	0.524	0.059
KTR N=46 patients (92 eyes)	Congenital/hereditary vs unknown/unspecified (N=11 vs. N=17)	CC density	2.33	-2.67 to 7.32	0.361	<0.001
		CMT (µm)	3.62	-9.42 to 16.66	0.587	<0.001
		DVPD (%)	-0.43	-4.79 to 3.93	0.847	0.034
		SVPD (%)	0.06	-4.31 to 4.43	0.979	0.016
		GCL+ (µm)	6.83	2.11 to 11.54	0.005	<0.001
		GCL++ (µm)	5.36	-1.29 to 12.00	0.114	0.080
		FAZ (µm²)	22.62	-56.25 to 101.48	0.574	<0.001
	Glomerular/immune vs unknown/unspecified (N=11 vs. N=17)	CC density	-2.65	-6.71 to 1.41	0.201	<0.001
		CMT (µm)	5.20	-5.68 to 16.08	0.349	<0.001
		DVPD (%)	-2.25	-4.92 to 0.42	0.099	0.034
		SVPD	-2.89	-5.65 to	0.040	0.016

Cohort	Contrast	Outcome	B	95% CI	p	Global p
		(%)		-0.13		
		GCL+ (μm)	0.71	-4.45 to 5.88	0.787	<0.001
		GCL++ (μm)	0.33	-6.42 to 7.08	0.923	0.080
		FAZ (μm²)	75.08	18.43 to 131.74	0.009	<0.001
	Tubulointerstitial/other vs unknown/unspecified (N=7 vs. N=17)	CC density	4.66	-0.04 to 9.35	0.052	<0.001
		CMT (μm)	20.26	9.98 to 30.55	<0.001	<0.001
		DVPD (%)	2.36	-1.94 to 6.67	0.283	0.034
		SVPD (%)	2.08	-2.24 to 6.41	0.345	0.016
		GCL+ (μm)	6.09	2.40 to 9.78	0.001	<0.001
		GCL++ (μm)	4.56	-1.76 to 10.88	0.157	0.080
		FAZ (μm²)	-30.50	-72.96 to 11.95	0.159	<0.001

Note: Values are adjusted regression coefficients from exploratory Gaussian GEE models with an exchangeable working correlation, clustered by patient and including both eyes. Analyses were performed separately within HD and KTR. Etiology models used unknown/unspecified etiology as the reference category and included etiologic categories with sufficient frequency in each cohort. Global p values are reported for the global effect of primary renal disease category. Results were not corrected for multiple testing and should be interpreted as hypothesis-generating.

Abbreviations*: CC, choriocapillaris; CI, confidence interval; CMT, central macular thickness; DVPD, deep vascular perfusion density; FAZ, foveal avascular zone; GCL+, ganglion cell layer plus inner plexiform layer; GCL++, ganglion cell complex; GEE, generalized estimating equation; HD, hemodialysis; KTR, kidney transplant recipients; OCT, optical coherence tomography; OCTA, optical coherence tomography angiography; SCT, subfoveal choroidal thickness; SVPD, superficial vascular perfusion density.*

Table S2. Stratified exploratory associations of medication use (users vs. non-users) and OCT/OCTA parameters.

Cohort	Factor	N	Outcome	B	95% CI	p
**HD** N=29 patients (56 eyes)	Calcium channel blocker use	10/19	GCL++ (µm)	-10.46	-16.05 to -4.86	<0.001
			SCT (µm)	-22.32	-42.89 to -1.74	0.033
	Central antihypertensive use	4/25	GCL++ (µm)	-10.81	-20.06 to -1.56	0.022
	Statin use	8/21	CC density	-0.66	-7.41 to 6.09	0.848
			CMT (µm)	3.41	-12.84 to 19.65	0.681
			DVPD (%)	1.01	-5.39 to 7.42	0.756
			FAZ (µm²)	44.56	-43.15 to 132.27	0.319
			GCL+ (µm)	-0.44	-4.91 to 4.03	0.846
			GCL++ (µm)	-5.34	-12.66 to 1.99	0.153
			SCT (µm)	15.49	-8.92 to 39.90	0.214
			SVPD (%)	0.82	-5.48 to 7.11	0.800
**KTR** N=48 patients (96 eyes)	ACEI/ARB use	7/41	GCL+ (µm)	-6.48	-12.62 to -0.35	0.038
			SCT (µm)	-27.30	-50.64 to -3.96	0.022
	Any antihypertensive medication	32/16	GCL+ (µm)	-4.65	-8.49 to -0.82	0.017
			GCL++ (µm)	-7.19	-14.10 to -0.28	0.041
	Beta blocker use	24/24	GCL++ (µm)	-8.36	-15.38 to -1.35	0.019
	Calcium channel blocker use	17/31	FAZ (µm²)	46.69	8.55 to 84.83	0.016
	Statin use	16/32	CC density	-0.04	-4.50 to 4.41	0.985
			CMT (µm)	5.27	-4.92 to 15.46	0.310
			DVPD (%)	0.13	-3.16 to 3.42	0.939
			FAZ (µm²)	-38.60	-82.87 to 5.67	0.087
			GCL+ (µm)	1.39	-2.59 to 5.37	0.494
			GCL++ (µm)	3.31	-3.51 to 10.13	0.341
			SCT (µm)	9.56	-11.41 to 30.53	0.372
			SVPD (%)	0.10	-3.28 to 3.49	0.951

Note: Values are adjusted regression coefficients from exploratory Gaussian GEE models with an exchangeable working correlation, clustered by patient and including both eyes. Analyses were performed separately within HD and KTR. ;for binary medication exposures, only the exposure-specific p value is shown. Results were not corrected for multiple testing and should be interpreted as hypothesis-generating.

***Abbreviations**: ACEI, angiotensin-converting enzyme inhibitor; ARB, angiotensin receptor blocker; CC, choriocapillaris; CI, confidence interval; CMT, central macular thickness; DVPD, deep vascular perfusion density; FAZ, foveal avascular zone; GCL+, ganglion cell layer plus inner plexiform layer; GCL++, ganglion cell complex; GEE, generalized estimating equation; HD, hemodialysis; HTN, hypertension; KTR, kidney transplant recipients; OCT, optical coherence tomography; OCTA, optical coherence tomography angiography; SCT, subfoveal choroidal thickness; SVPD, superficial vascular perfusion density.*

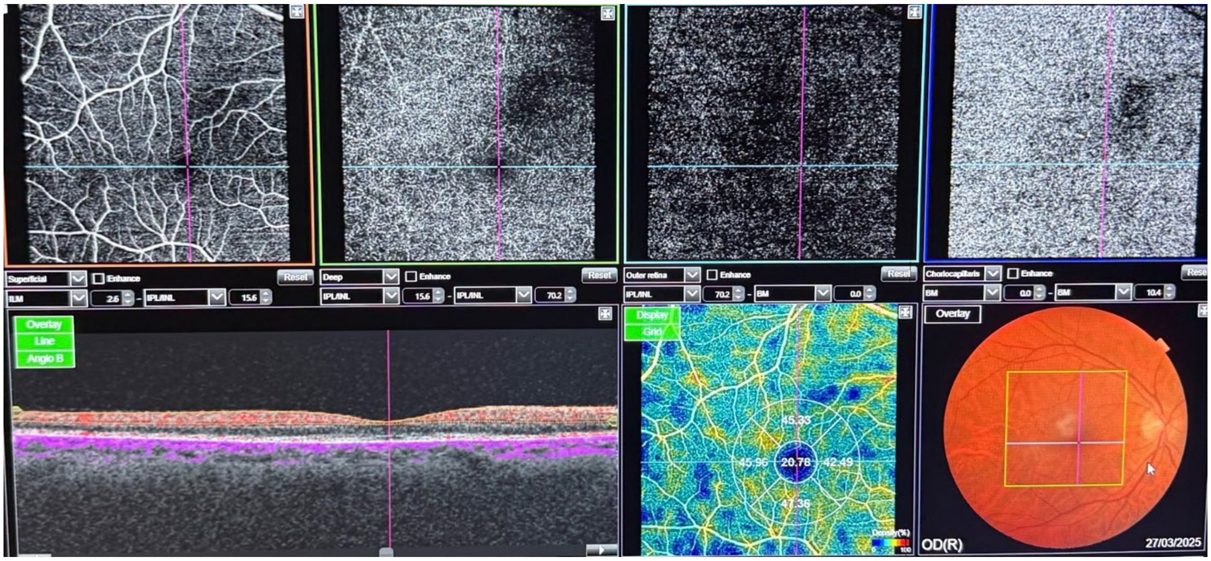

Supplementary Figure S1. Representative OCTA acquisition from a hemodialysis patient obtained using swept-source OCT angiography (DRI OCT Triton, Topcon, 2015). The figure includes en face images of the superficial vascular plexus, deep vascular plexus, outer retinal slab, and choriocapillaris, together with the corresponding structural OCT B-scan and segmentation boundaries. The image illustrates the acquisition and segmentation protocol used for quantitative retinal and microvascular assessment.
